# Formulation of Antimicrobial Tobramycin Loaded PLGA Nanoparticles via Complexation with AOT

**DOI:** 10.3390/jfb10020026

**Published:** 2019-06-13

**Authors:** Marcus Hill, Richard N. Cunningham, Rania M. Hathout, Christopher Johnston, John G. Hardy, Marie E. Migaud

**Affiliations:** 1 School of Pharmacy, Queen’s University Belfast, Belfast BT7 1NN, UK; mhill11@qub.ac.uk (M.H.); rcunningham09@qub.ac.uk (R.N.C.); christopher.johnston@qub.ac.uk (C.J.); 2 Department of Pharmaceutics and Industrial Pharmacy, Faculty of Pharmacy, Ain Shams University, Cairo 11566, Egypt; rania.hathout@pharma.asu.edu.eg; 3 Department of Chemistry, Lancaster University, Lancaster, Lancashire LA1 4YB, UK; 4 Materials Science Institute, Lancaster University, Lancaster, Lancashire LA1 4YB, UK; 5 USA Mitchell Cancer Institute, University of South Alabama, Mobile, AL 36604, USA

**Keywords:** biomedical applications, colloids, drug delivery systems, nanoparticles, antimicrobial

## Abstract

Tobramycin is a potent antimicrobial aminoglycoside and its effective delivery by encapsulation within nanoparticle carriers could increase its activity against infections through a combination of sustained release and enhanced uptake. Effective antimicrobial therapy against a clinically relevant model bacteria (*Pseudomonas aeruginosa*) requires sufficient levels of therapeutic drug to maintain a drug concentration above the microbial inhibitory concentration (MIC) of the bacteria. Previous studies have shown that loading of aminoglycoside drugs in poly(lactic-co-glycolic) acid (PLGA)-based delivery systems is generally poor due to weak interactions between the drug and the polymer. The formation of complexes of tobramycin with dioctylsulfosuccinate (AOT) allows the effective loading of the drug in PLGA-nanoparticles and such nanoparticles can effectively deliver the antimicrobial aminoglycoside with retention of tobramycin antibacterial function.

## 1. Introduction

*Pseudomonas aeruginosa* is an aerobic gram negative bacterium which can survive in a variety of environments [1]. *P. aeruginosa* infections in immune competent patients tend to arise through physical trauma or surgical complications [2,3]. The majority of serious infections involve patients in which the normal functioning of the immune system has been compromised (e.g., AIDS and cystic fibrosis [4,5]), or cases involving complications caused by the administration of broad spectrum antibiotics which can disrupt the normal protective mucosal flora [6]. *P. aeruginosa* infection is increasingly prevalent and is responsible for 16% of nosocomial pneumonia infections, 10% of bloodstream infections and 8% of surgical wound infections reported [7]. 

There is a market need for systems capable of the controllable delivery of hydrophilic drugs [8]. Many drugs have been shown to achieve greater therapeutic efficacy through loading within nanoparticulate drug delivery systems based on their potential for precise targeting and sustained release [9,10,11]. However, this formulation remains difficult to achieve for highly polar, water-soluble drugs. While the hydrophilicity of a drug can offer advantages including improved bioavailability and absorption [12], this can lead to poor loading within polymer-based nanoparticles if interactions between the drug and the polymer are weak [13]. 

Yet, the formulation of antibiotic-loaded nanoparticles has been applied to address a variety of diseases [14,15,16], with such formulations showing a number of advantages over conventional administration (including enhanced antibacterial effect and targeting) [17]. The targeted delivery and sustained release of the drug over time permit specific delivery to the diseased site, allowing for the reduction in the frequency of dosing and reduction in the off-site toxicity experienced with certain drugs [18].

**Figure 1:**
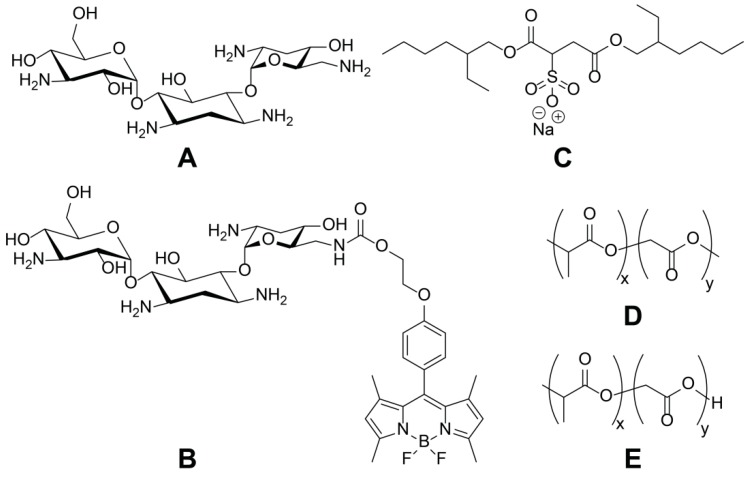
Chemical structures investigated in this report. (**A**) Tobramycin; (**B**) Fluorescent tobramycin derivative; (**C**) dioctylsulfosuccinate (AOT); (**D**) PLGA 503 ester terminated; (**E**) PLGA 502H acid terminated.

Aminoglycosides are a class of drug molecules which are generally prescribed for the treatment of bacterial infections, e.g., to the lungs to treat respiratory conditions [19,20]. This family of highly polar drugs shares a similar chemical scaffold although subtle spatial and functional group changes lead to differences in the therapeutic activity of the drug [19]. Tobramycin (Figure 1) is most commonly prescribed for the treatment of infections with *P. aeruginosa* [21]. Its antibacterial activity is mediated through binding to the 30 s ribosomal subunit in gram negative bacterial strains [22], while rapid efflux is accomplished by phosphorylation [23,24,25]. The incorporation of aminoglycoside drugs within PLGA-based carriers has previously proven challenging [26] due to the lack of complementary polar groups on the backbone of PLGA to interact with the aminoglycosides (i.e., weak polymer-drug interactions) [27]. Furthermore, non-aqueous solvents are commonly required for the formation of the nanoparticles. These often prove to be poor solvents for the aminoglycosides and therefore a limiting factor in the formulation process.

Alternative solvents can be employed to improve the loading of hydrophilic drugs within hydrophobic polymers (e.g., supercritical fluids) [28]. However, this relies upon the co-dissolution of the drug and the polymer within the supercritical fluid which remains challenging [29]. 

Poly-lactic-co-glycolic acid (PLGA) has been utilised for a variety of sustained release drug delivery systems [30,31,32], for example, the aminoglycoside antibiotic gentamicin [33]. Studies have shown that the incorporation of such drugs in PLGA nanoparticles can offer enhanced therapeutic benefit in biological models of disease [34]. However, the overall loading of the hydrophilic drugs can limit the therapeutic benefit because of weak polymer–drug interactions [35,36]. This observation motivates the investigation of methods that can increase polymer–drug interactions with the view of enhancing the level of drug-loading within the nanoparticles. Chemical modifications of the drug can enhance polymer–drug interactions while also retaining the activity of the drug [37]. An alternative approach is the co-formulation with a surfactant [38], which was used successfully for the preparation of liposomal formulations of tobramycin to treat infections with *P. aeruginosa* [39,40,41,42,43].

The anionic surfactant AOT is FDA approved for human therapy [44] and has been used to extract various hydrophilic drugs and proteins into organic media [45,46,47]. To facilitate the detection of non-chromophoric drugs in the aqueous media, we prepared a fluorescent tobramycin derivative, and here we report the results of our studies investigating the co-formulation of non-chromophoric tobramycin and AOT, enabling its encapsulation within, and release from, PLGA nanoparticles (including a comparison of the constitutional and electronic physical descriptors of tobramycin, the fluorescent tobramycin derivative and their AOT-complexes [48,49]). The PLGA-based nanoparticles delivered tobramycin from the PLGA nanoparticles to the clinically relevant model bacteria (*P. aeruginosa*) at levels above the microbial inhibitory concentration (MIC) of the bacteria.

## 2. Results and Discussion

### 2.1. Tobramycin Formulation

The uptake efficiency of tobramycin by PLGA is governed by drug–polymer interactions. We evaluated two forms of PLGA: PLGA RG502H and PLGA RG503. PLGA RG502H is more hydrophilic and was previously shown to trap hydrophilic drugs more efficiently [33]. PLGA nanoparticle production has been the subject of many investigations and excellent reviews [50,51]. In this study we formulated nanoparticles using water-in-oil-in-water (w/o/w) emulsion [52] and solid-in-oil-in-water (s/o/w) emulsion [53] methodologies, and their properties and tobramycin loading are reported in Table 1. Both methodologies resulted in loading of tobramycin within the nanoparticles (albeit relatively low concentrations of drug in the particles). We observed that the w/o/w formulation methodology resulted in the generation of smaller particles (determined by Dynamic Light Scattering, DLS) than the s/o/w methodology, and that the s/o/w methodology also resulted in higher levels of tobramycin loading; and as expected the nanoparticles generated from the more hydrophilic PLGA derivative (RG502H) had a moderately higher zeta potential and drug loading (although this was not statistically significant).

**Scheme 1:**
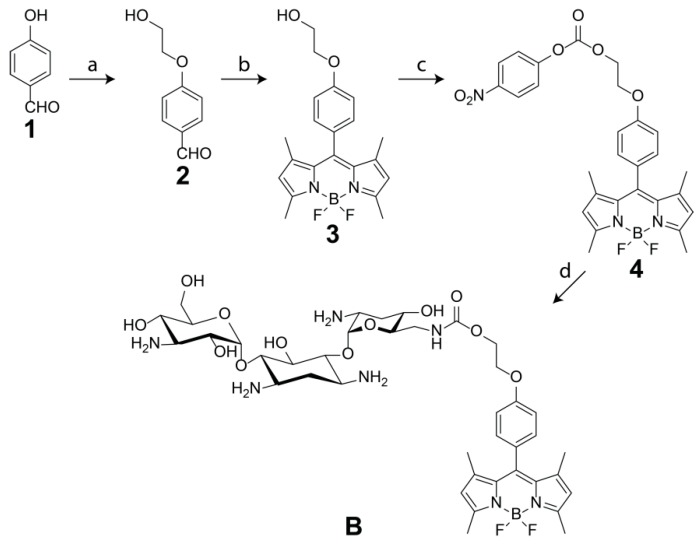
Synthesis of a fluorescent tobramycin derivative.

**Figure 2:**
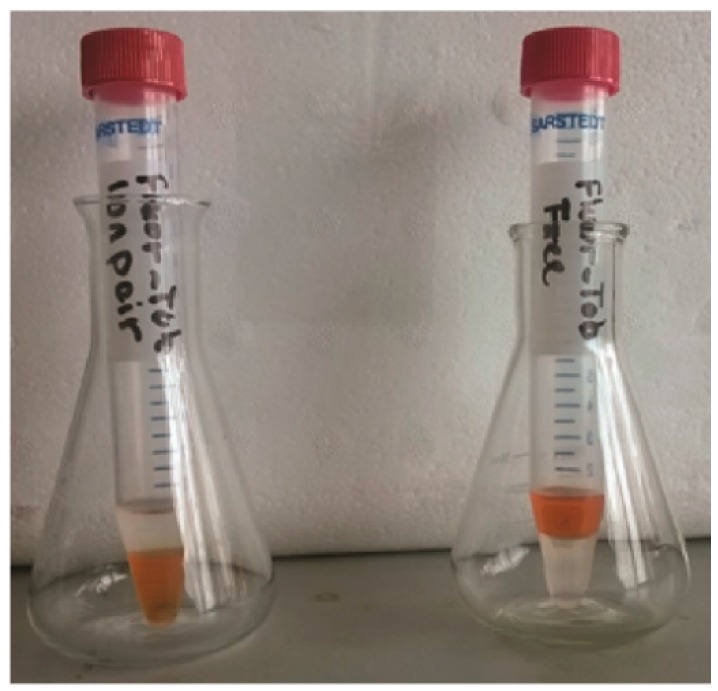
Organic extraction of the fluorescent derivative of tobramycin in the presence (left) and absence (right) of AOT: top layer water, bottom layer dichloromethane.

With a view to increase the loading of tobramycin within the PLGA nanoparticles, we investigated the co-formulation of the drug with a surfactant (AOT) that has previously been used to increase the loading of other hydrophilic drugs within PLGA nanoparticles. This was achieved by increased lipophilicity of the drug-AOT complexes generated through ionic interactions; in this case the anionic sulfonate of the AOT and the cationic amine of tobramycin) [54,55,56]. To assess the lipophilicity of the tobramycin-AOT complexes, the extraction of tobramycin into organic solvents was assessed by a variety of methods. A fluorescent tobramycin derivative (B) was synthesized (Scheme 1, and Supplementary Information Figures S1–S13) which facilitated the visual observation of its extraction into the aqueous layer of a dichloromethane-water bilayer (Figure 2).

The physical descriptors (constitutional and electronic) of tobramycin, the fluorescent tobramycin derivative and their AOT-complexes are calculated (Table 2). The selected descriptors were molecular weight, total polar surface area, number of hydrogen bond donors, number of hydrogen bond acceptors, molecular globularity, molecular flexibility and LogP (O/W) [48,49].

The significant changes in molecular descriptors upon conjugation of the fluorophore to tobramycin (particularly total polar surface area, molecular globularity, molecular flexibility and the LogP (O/W) value) meant that we did not carry out encapsulation or release studies with the fluorescent derivative as it would lead to inaccurate predictions of the encapsulation and release of the non-fluorescent tobramycin, that would be clinically relevant. Consequently, tobramycin extraction into a selection of non-aqueous solvents was quantified by reaction of tobramycin with ortho-phthaldialdehyde and 2-mercaptoethanol and subsequent fluorescence spectroscopy [57] with results displayed in Table 3. In the light of this data and literature precedent [58], we used dichloromethane for the remaining loading experiments. As expected, we observed that the molar ratio of AOT:tobramycin played a role in the extraction of tobramycin into dichloromethane, with complete extraction above a molar ratio of 0.1 AOT:tobramycin (Figure S14).

**Figure 3:**
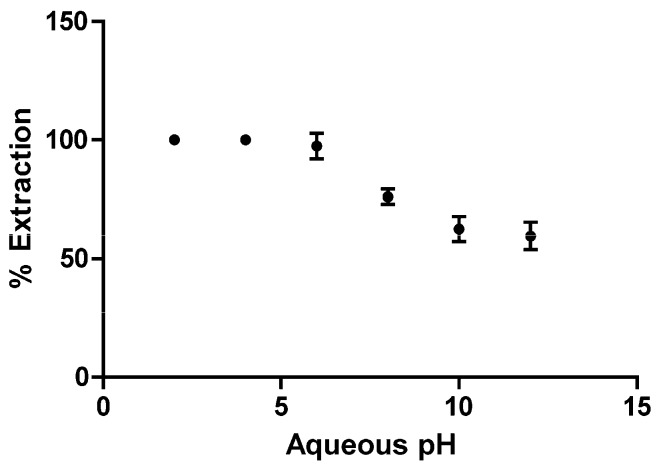
Effect of pH on the extraction of tobramycin into dichloromethane by AOT.

It was also expected that there would be a pH dependence on the extraction of tobramycin into dichloromethane. The sulfonic acid of AOT is deprotonated at most pH values, and we observed that at low pH values (pH 2 and 4), the amines displayed on tobramycin were fully protonated and the tobramycin was completely partitioned in the dichloromethane (Figure 3). Above pH 5 we observed some tobramycin remained in the aqueous phase as not all the amines were fully protonated, with only 59% of the drug extracted into the dichloromethane phase at pH 12.

To assess if the increased solubility of tobramycin in non-aqueous solvents (Table 3, Figure 2 and Figure 3) and the changes in molecular descriptors (particularly molecular globularity, molecular flexibility and the LogP (O/W), in Table 2) correlated to increased uptake of tobramycin within the PLGA nanoparticles [50,59,60], we prepared solutions of PLGA nanoparticles from O/W emulsions (with the oil phase composed of solutions of PLGA and tobramycin:AOT complexes in dichloromethane). In contrast to the experiments undertaken in the absence of AOT, the PLGA derivative had no statistically significant effect on the particle properties or tobramycin loading (Table 4). The particle size and zeta potential of the nanoparticles were observed to increase as the concentration of PLGA in the emulsions increased. The zeta potential values of the nanoparticles appeared to be dependent on the PLGA derivative, with the PLGA RG502H (carboxyl terminated PLGA chains) particles having lower zeta potential values than PLGA RG503 (methyl ester terminated PLGA chains), although this was not statistically significant. High loading was observed for all the formulations being tested (>89%). With a view to sustaining the release of the tobramycin from the particles (with minimal burst release from drug close to the surface of the particles) we chose to use the nanoparticles derived from emulsion formulations containing 30 mg of PLGA RG502H for subsequent experiments.

### 2.2. In Vitro Tobramycin Release Studies

**Figure 4:**
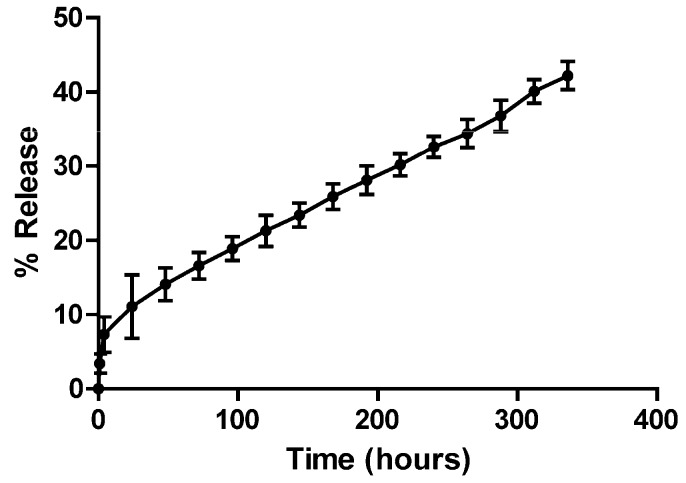
Release profile of PLGA RG502H nanoparticles entrapped with tobramycin loaded AOT reverse micelles at 37 °C in PBS.

As the treatment of chronic infections with *P. aeruginosa* generally requires prolonged exposure to the antimicrobial for efficient therapy, the sustained release properties of the tobramycin-AOT loaded PLGA RG502H nanoparticles (prepared from emulsions containing 30 mg of PLGA RG502H) were tested (Figure 4). The slightly high rate of tobramycin release over the first few hours was ascribed to the release of tobramycin located close to the surface of the nanoparticles (ca. 10% of the total load of tobramycin); however, after this initial release from the surface, the internal tobramycin was released at a much slower rate and this over a period of days, with less than half the total payload delivered in 2 weeks (Figure 4) offering potential for use of less drug and lower dosing frequency which would be expected to enhance patient compliance (i.e., economic, environmental, health and societal impacts).

**Figure 5:**
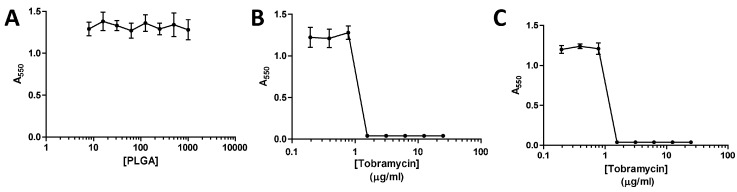
MIC analysis. (**A**) Unloaded nanoparticle control; (**B**) Free tobramycin; (**C**) Tobramycin loaded nanoparticles.

The effect of tobramycin release from the PLGA RG502H nanoparticles loaded with tobramycin-AOT complexes was tested against *P. aeruginosa* (Figure 5). Unloaded PLGA RG502H nanoparticles showed no activity against *P. aeruginosa* (Figure 5A), whereas the free tobramycin showed dose dependent toxicity towards the *P. aeruginosa* (Figure 5B), with the MIC of the tobramycin at 1.25 µg/mL. Importantly, the PLGA RG502H nanoparticles loaded with tobramycin-AOT complexes showed activity in a dose dependent manner (Figure 5C). The MIC of the tobramycin encapsulated in the nanoparticles was shown to be 1.25 µg/mL, identical to that of free tobramycin, confirming that no loss of activity was detected following encapsulation. The full release of tobramycin would be achieved over days in the absence of cells (Figure 5); however, once internalized in the cells, the release of tobramycin would be likely to be enhanced by enzymatic degradation of the PLGA [61].

Researchers based in industry and academia have invested significant effort into the development of active ingredient delivery systems. Systems that deliver their payloads at a predetermined rate and control the level of active ingredient within target tissues above the minimum effective level for prolonged periods help to reduce the dosing frequency (and concomitantly problems with patient compliance), which offer economic, environmental, health and societal impacts. There is a market need for delivery systems with sustained release of polar antimicrobials capable of eradicating bacterial growth in patients with weak immune systems [62,63,64,65,66,67,68]. Here we describe one method of loading high levels of antimicrobials in PLGA nanoparticles and demonstrate their efficacy against *P. aeruginosa* in vitro, using transient drug–polymer interactions for encapsulation and slow-release, and we envisage such systems to have potential for the treatment of respiratory infections [69,70].

## 3. Materials and Methods

### 3.1. Materials

Tryptone, sodium chloride (bacteriological grade) and yeast extract were purchased from Oxoid Ltd, Basingstoke, Hampshire, England. Formvar carbon films on copper were purchased from Agar scientific (Stansted, UK). The polymers: PLGA Resomer RG502H (50:50 lactide:glycolide, acid terminated); PLGA Resomer RG503 (50:50 lactide:glycolide, ester terminated); Poly(vinyl alcohol) (PVA), 87–89% hydrolysed with molecular weight 13–23 KDa; and all other consumables were purchased from Sigma-Aldrich (Gillingham, UK) unless otherwise stated.

### 3.2. Preparation of the Fluorescent Tobramycin Derivative and Its Use for Visualisation of Bilayer Extraction Experiments

#### 3.2.1. Synthesis of 4-(2-hydroxyethoxy)benzaldehyde

4-Hydroxybenzaldehyde (**1**, Scheme 1) (1.50 g, 12.28 mmol, 1.0 eq), 2-bromoethanol (1.31 mL, 18.42 mmol, 1.5 eq) and K_2_CO_3_ (5.10 g, 36.85 mmol, 3.0 eq) were solubilized in DMF (15 mL). The reaction mixture was stirred for 6 h at 100 °C and then poured into water (15 mL) and extracted with chloroform (3 × 25 mL). The organic layers were combined and dried over Na_2_SO_4_. The solvent was removed under reduced pressure and purified using biotage column chromatography (Hexane/EtOAc) 6:4) to give 2.15g (93%) of 4-(2-hydroxyethoxy)benzaldehyde as a colorless oil. 1H NMR (400 MHz, CDCl3): δ ppm 9.89 (1H, s, HC=O), 7.85 (2H, d, J = 9.3 Hz, Ar), 7.03 (2H, d, J = 8.7 Hz, Ar), 4.18 (2H, t, J = 9.0 Hz, OCH2CH2OH), 4.02 (2H, t, J = 9.3 Hz, OCH2CH2OH).13C NMR (125 MHz): δ ppm 190.9 (HC=O), 163.7 (Ar), 132.0 (Ar), 130.2 (Ar), 114.9 (Ar), 69.6 (OCH2CH2OH), 61.2 (OCH2CH2OH), (ES) m/s: C9H10O3: Calculated [M+H]^+^ 189.0528, actual [M+H]^+^ found 189.0519.

#### 3.2.2. Synthesis of (3, 5-dimethyl-1H-pyrrol-2-yl)-(tetramethyl-4, 4-difluoro-4-bora-3a, 4a-diaza-indacene) methyl] phenoxy] ethanol

4-(2-Hydroxyethoxy) benzaldehyde (**2**, Scheme 1) (1.50 g, 0.01 mol, 1.0 eq) and 2-4-dimethylpyrrole (1.95 mL, 0.02 mol, and 2.0 eq) were solubilised in dry DCM (200 mL) under an atmosphere of N_2_. TFA (cat) added to the solution and the reaction mixture was left stirring at room temperature overnight. After disappearance of the aldehyde monitored using TLC, a solution of DDQ (2.00 g, 0.01 mol, and 1.0 eq) in dry DCM (5 mL) was added and stirring was continued for 30 min at room temperature. To the mixture was then added triethylamine (9.60 mL, 0.07 mmol, 7.6 eq) and left stirring for 15 min, after which BF_3_.OEt_2_ (9.60 mL, 0.08 mmol, 8.6 eq) was added drop wise at 0 °C. The stirring was continued overnight; then the solution was concentrated under reduced pressure and purified using biotage column chromatography (DCM/EtOAc) 0–10%) to yield 0.96 g (25%) of the fluorophore as fine orange needles. 1H NMR (400 MHz, CDCl3): δ ppm 7.18 (2H, d, J = 8.0 Hz, Ar), 7.03 (2H, d, J = 8.8 Hz, Ar), 5.98 (2H, s, 2 × C=CHC), 4.15–4.14 (2H, m, OCH2CH2OH), 4.02 (2H, t, J = 4.4 Hz, OCH2CH2OH), 2.55 (6H, s, 2× CH3CN) 1.43 (6H, s, 2 × CH3C=C).13C NMR (125 MHz): δ ppm 159.3 (Ar), 155.3 (Ar), 143.1 (Ar), 141.7 (Ar), 131.8 (Ar), 129.3 (Ar), 127.4 (Ar), 121.1 (Ar), 115.1 (Ar), 69.3 (OCH2CH2OH), 61.3 (OCH2CH2OH), 14.5 (2 × CH3CN and 2 × CH3C=C). 19F NMR (376MHz): δ ppm −143.3 ppm (B-F2, q, J = 31.3 Hz) (ES) m/s: C21H24BF2N2O2: Calculated [M+H]^+^ 385.1899, actual [M+H]^+^ found 385.1911.

#### 3.2.3. Synthesis of tetramethyl-4,4-difluoro-4-bora-3a,4a-diazaindacene)methyl]phenoxy]ethyl (4-nitrophenyl) carbonate

To a solution of (**3**, Scheme 1) (0.50 g, 1.30 mmol, 1.0 eq) in dry DCM (15 mL) was added 4-nitrobenzyl chloroformate (0.34 g, 1.69 mmol, 1.3 eq) and TEA (0.36 mL, 2.60 mmol, 2.0 eq). The reaction mixture was left stirring for 2 h or until complete disappearance of the starting compound by TLC. The reaction mixture was concentrated and purified using biotage column chromatography (DCM/MeOH) 0–5%) to yield 300.3 mg (42%) of PNP-BODIPY as orange needles. ^1^H NMR (400 MHz, CDCl_3_): δ ppm 8.30 (2H, d, J = 9.2 Hz, Ar), 7.42 (2H, d, J = 8.8 Hz, Ar), 7.21 (2H, d, J = 8.4 Hz, Ar), 7.05 (2H, d, J = 8.4 Hz, Ar), 5.98 (2H, s, 2 × C=CH-C), 4.69 (2H, t, J = 4.5 Hz, OCH2CH2OC=O), 4.34 (2H, t, J = 4.5 Hz, OCH2CH2OC=O), 2.55 (6H, s, 2 × CH3-C-N), 1.43 (6H, s, 2 × CH3-C=C).13C NMR (125 MHz): δ ppm 158.70 (O=C=O), 155.5 (Ar), 155.4 (Ar), 152.5 (Ar), 145.6 (Ar), 143.0 (Ar), 141.4 (Ar), 131.8 (Ar), 129.5 (Ar), 128.0 (Ar), 125.4 (Ar), 125.0 (Ar), 122.2 (Ar), 121.2 (2 × C=CH-C), 115.6 (Ar), 115.2 (Ar), 67.3 (OCH2CH2OC=O), 65.5 (OCH2CH2OC=O), 14.6 (2 × CH3CN & 2 × CH3C=C). ^19^F NMR (376MHz): δ −146.2 (B-F2, q, J = 31.6 Hz), (ES) m/s: C28H27BF2N3O6: Calculated [M+H]^+^ 550.1950, actual [M+H]^+^ found 550.1947.

#### 3.2.4. Synthesis of the Fluorescent Tobramycin Derivative (B) Used for Visualizing Bilayer Extraction

Tobramycin (free base) (45 mg, 0.091 mmol, 1.00 eq) was solubilized in anhydrous DMF (5.0 mL) with the assistance of gentle heating, then the solution was allowed to cool to room temperature. To the solution was added (**4**, Scheme 1) (50 mg, 0.095, 1.05 eq) and Hunig’s base (44 µL, 0.364 mmol, 4.0 eq) and left stirring overnight protected from light by a layer of aluminium foil. The reaction mixture was concentrated under high vacuum then purified using silica column chromatography using an eluent of (DCM/MeOH/NH_4_OH) 6:3:1) to yield 13.6 mg (17%) of the titled compound as an orange solid. ^1^H NMR (400 MHz, D_2_O): δ ppm 8.04 (2H, d, J = 8.5 Hz, Ar), 7.42 (1H), 7.2 (1H), 7.0 (2H), 6.84 (2H, d, J = 8.5 Hz, Ar), 6.74 (2H), 5.78 (2H), 5.39 (2H), 4.21 (2H), 4.06–3.15 (17H, m), 2.65 (3H), 2.44 (2H), 2.30–2.05 (6H, m), 1.86 (2H), 1.62–1.31 (4H, m), 1.17–0.87 (6H, m), 0.72–0.56 (3H, m). ^19^F NMR (376 MHz): δ −146.2 (B-F_2_, q, J = 32.0Hz), (ES) m/s: C_40_H_59_BN_7_O_12_F_2_: Calculated [M+H]^+^ 878.4305, actual [M+H]^+^ found 878.4283.

#### 3.2.5. Visualization of the Extraction of the Fluorescent Tobramycin Derivative

Stock solutions of fluorescent tobramycin (1.5 mg/mL) in deionized water and AOT (7.1 mg/mL) in DCM were prepared. The extraction of the fluorescent tobramycin derivative from the aqueous phase (2 mL) into the organic phase (2 mL) with/without AOT was assessed by mixing the phases together at 600 rpm for 3 h after which the phases were separated by centrifugation at 4400 rpm enabling visualization of the tobramycin in the aqueous or organic layer.

### 3.3. Calculating the Main Physical Descriptors of Tobramycin Derivatives and AOT Complexes Thereof

The physical descriptors (constitutional and electronic) of tobramycin and its AOT-complex were calculated. The selected descriptors were the total polar surface area, number of hydrogen bond donors, number of hydrogen bond acceptors, molecular globularity, molecular flexibility, LogP (O/W) and the molecular weight. The descriptors were calculated using MOE version 2014.0901 (Chemical Computing Group Inc., Montreal, Canada) after constructing the two investigated drugs utilising the builder tool in the same software to generate the 3D structures of the investigated drugs from their isomeric SMILES obtained from The PubChem Project^®^ [48,49].

### 3.4. Analytical Methodology for the Detection of Tobramycin

Reagent A consisting of 80 mg of ortho-phthaldialdehyde in 1 mL of 95% ethanol and reagent B containing 200 µL of boric acid (pH 9.7, 0.4 M), 400 µL β-mercaptoethanol and 200 µL of diethyl ether were mixed. Serial dilutions of tobramycin were prepared in boric acid (pH 9.7, 0.4 M). One hundred µL of each tobramycin standard was added to 100 µL of the reagent mixture. The plate was read by fluorescence spectroscopy at λ_ex_ 360 nm, λ_em_ 460 nm, respectively. The calibration curve is displayed in Figure S15.

### 3.5. W/O/W and S/O/W Preparation of PLGA Nanoparticles Entrapping Tobramycin

Tobramycin loaded PLGA nanoparticles were prepared through two separate methodologies. For the water in oil in water (W/O/W) methodology, a primary emulsion was formed by dissolving 3 mg of tobramycin in 0.5 mL of water followed by emulsification by sonication at 40 watts in 2 mL of DCM containing 50 mg of PLGA RG502H or PLGA RG503. The resulting W/O emulsion was added to 10 mL of PVA (2.5% in PBS buffer, pH 7.4) followed by further sonication to form the final W/O/W emulsion. For the solid in oil in water (S/O/W) methodology, 3 mg of tobramycin was dissolved in 100 µL of water and added to 2 mL of acetone containing 50 mg of PLGA RG502H or 503. The resulting S/O phase was added to 10 mL of PVA (2.5% in 25 mM PBS buffer) to form the final S/O/W formulation. Organic solvent was removed under vacuum at room temperature. Both formulations were centrifuged at 20,000 g and washed in PBS three times by centrifugation resuspension cycles. Results are presented as mean ± S.D, N = 3.

### 3.6. Extraction of Tobramycin into Organic Solvents by AOT

Stock solutions of tobramycin (1.5 mg/mL pH 4) and surfactant AOT (7.1 mg/mL) were prepared in double distilled water and a range of organic solvents respectively. Two mL of tobramycin stock solution was added to an equal volume of AOT stock solution in each of the organic solvents under study and the two solutions were mixed at 600 rpm for 3 h. The phases were separated by centrifugation at 4400 rpm for 10 min and the aqueous phase was collected and monitored for the presence of tobramycin. The fluorescence intensity of the aqueous phase was monitored at λ_ex_/λ_em_ 360/460 nm and compared to a standard curve of tobramycin base including surfactant AOT at a concentration of 2 mg/mL. Results are presented as mean ± S.D, N = 3.

To assess the effect of molar ratio of AOT to tobramycin on the extraction process, the molar ratio of surfactant AOT to tobramycin was varied. A stock solution of tobramycin was prepared in double distilled water (DDW) (1.5 mg/mL, pH 4) and 2 mL was added to a stock solution of AOT in DCM at varying concentrations. The resultant suspension was stirred at 600 rpm for 3 h. The phases were separated by centrifugation at 4400 rpm and the concentration of tobramycin in the aqueous phase was determined as described previously. Results are presented as mean ± S.D, N = 3.

To assess the effect of formulation pH on the extraction process stock, solutions of tobramycin were prepared (1.5 mg/mL) and the pH was adjusted to 2, 4, 6, 8, 10 and 12. 2 mL of each stock solution of tobramycin was then added to 2 mL of surfactant AOT (7.1 mg/mL) in DCM. The solutions were mixed at 600 rpm for 3 h. The phases were separated by centrifugation at 4400 rpm and the concentration of tobramycin in the aqueous phase was determined as described previously. Results are presented as mean ± S.D, N = 3.

### 3.7. Preparation of PLGA Nanoparticles Loaded with Tobramycin-AOT Complexes

Equal volumes of tobramycin base (1.5 mg/mL in water adjusted to pH 4) and AOT (7.1 mg/mL in dichloromethane) (1:5 molar ratio) were mixed by stirring for 3 h at 600 rpm at room temperature. After incubation the aqueous and organic phases were separated by centrifugation for 5 min at 4400 rpm, and the dichloromethane removed by evaporation yielding a very viscous oil. Three mg of the viscous oil was dissolved in DCM (2 mL) with 10–50 mg of either PLGA RG502H or PLGA RG503. The DCM was added to 10 mL of PVA (2.5% in PBS buffer pH 7.4) followed by sonication at 40 watts to form an O/W emulsion. DCM was removed under vacuum at room temperature. Formulations were centrifuged at 20,000 g and washed with PBS three times by centrifugation-resuspension cycles. The amount of tobramycin entrapped in the nanoparticles was assessed by determination of the tobramycin content in the supernatant collected after nanoparticle formulation.

### 3.8. Dynamic Light Scattering (DLS) and Zeta Potential Measurements

DLS and zeta potential measurements were performed using a Malvern zetasizer (Nano ZS; Malvern instruments, Malvern, UK). Each sample was recorded in triplicate (10 runs each). The average of three separate samples was determined and are presented as mean ± S.D, N = 3.

### 3.9. Release of Tobramycin from PLGA Nanoparticles Loaded with Tobramycin-AOT Complexes

Drug release was quantified by incubating the nanoparticles in dialysis membranes with a 10,000 Da MWCO at 37 °C under agitation. The release of the tobramycin was quantified by incubating 3 mg of nanoparticles in 1 mL of PBS in the donor compartment with 5 mL of PBS in the receiver compartment. At each time point, the PBS solution was collected and replaced with fresh PBS release medium. The concentration of tobramycin in the aqueous phase was determined as described previously. Results are presented as mean ± S.D, N = 3.

### 3.10. Antimicrobial Activity against P. aeruginosa

Broth micro-dilution tests were performed according to NCCLS guidelines. Serial two-fold dilutions of tobramycin (from a stock solution which had been sterile filtered through a 0.22 µm filter) in 100 µL of Luria Bertani (LB) broth were performed on a 96-well plate in the range 0–25 µg tobramycin/mL for the drug loaded nanoparticles, free tobramycin and the blank nanoparticles as negative control. The actively growing cultures were diluted to an optical density reading of 0.3 (A_550_) to give a starting inoculum of 2 × 10^5^ CFU/mL. One hundred µL of the starting inoculum (2 × 10^5^ CFU/mL) was added to each well of the plate and incubated aerobically at 37 °C for 24 h. Following incubation, the cell viability was determined by reading the plate absorbance at 550 nm. Results are presented as mean ± S.D, N = 3.

### 3.11. Statistical Analysis

Data was analysed with Graphpad Prism (San Diego, CA, USA). Experiments were performed in triplicate and the mean value reported ± S.D. 

## 4. Conclusions

There is a market need for the development of drug delivery systems and sustained release of polar antimicrobials capable of eradicating bacterial growth in patients with weak immune system [62,63,64,65,66,67,68]. Here we describe one method of loading high levels of antimicrobials in PLGA nanoparticles and demonstrate their efficacy against *P. aeruginosa* in vitro, using transient drug-polymer interactions for encapsulation and slow-release.

## Figures and Tables

**Table 1:** Particle properties and tobramycin loading achieved with PLGA RG502H and RG503 nanoparticles with the S/O/W and W/O/W formulation strategies.

PLGA Derivative	Formulation	DLS Particle Size (nm)	PDI	Zeta Potential (mV)	Tobramycin Loading in PLGA Nanoparticles (µg/mg)	% Loading
RG503	w/o/w	267.4 ± 5.3	0.14 ± 0.02	−7.8 ± 2.2	2.7 ± 0.4	4.5 ± 0.7
RG502H	w/o/w	259.8 ± 6.8	0.18 ± 0.04	−9.6 ± 3.2	3.9 ± 0.3	6.5 ± 0.5
RG503	s/o/w	345.8 ± 17.2	0.16 ± 0.08	−8.2 ± 2.8	3.4 ± 0.7	5.7 ± 1.2
RG502H	s/o/w	364.8 ± 22.4	0.22 ± 0.11	−9.4 ± 3.3	4.4 ± 0.6	7.3 ± 1.0

**Table 2:** The physical descriptors (constitutional and electronic) of tobramycin, the fluorescent tobramycin derivative and their AOT-complexes.

Species	Molecular Weight (g/mol)	Total Polar SA	No. H Bond Acceptors	No. H Bond Donors	Molecular Globularity	Molecular Flexibility	LogP (O/W)
Tobramycin derivative	467.520	420.078	14	10	0.123	9.277	−6.412
Fluorescent tobramycin derivative	878.756	694.379	15	10	0.163	12.913	−1.894
AOT-Tobramycin complex	912.085	694.379	16	10	0.252	29.588	−1.342
AOT-Fluorescent tobramycin complex	1323.321	852.276	17	10	0.218	29.376	−3.176

**Table 3:** Extraction of tobramycin into non-aqueous solvents in the absence/presence of AOT.

Solvent	Dielectric Constant	% Extraction in the Absence of AOT	% Extraction in the Presence of AOT
2-butanol	17.26	0 ± 0	90.3 ± 5.7
Chloroform	4.81	0 ± 0	100 ± 0
Dichloromethane	8.93	0 ± 0	100 ± 0
Ethyl acetate	6.02	0 ± 0	100 ± 0
Toluene	2.38	0 ± 0	100 ± 0

**Table 4:** Properties and tobramycin uptake of PLGA RG502H and PLGA RG503 nanoparticles.

PLGA Derivative	Mass of PLGA (mg)	Particle Size (nm)	PDI	Zeta Potential (mV)	Tobramycin Loading in PLGA Nanoparticles (µg/mg)	% Loading
RG502H	10	229.4 ± 21.6	0.23 ± 0.09	−11.2 ± 3.1	274.2 ± 9.6	91.4 ± 3.2
RG502H	20	434.5 ± 52.5	0.42 ± 0.11	−11.8 ± 3.2	96.4 ± 2.9	96.4 ± 2.9
RG502H	30	469.2 ± 57.2	0.39 ± 0.06	−12.2 ± 2.8	57.5 ± 1.3	95.8 ± 2.2
RG503	10	295.6 ± 46.4	0.26 ± 0.10	−8.5 ± 3.3	269.4 ± 7.2	89.8 ± 2.4
RG503	20	437.2 ± 49.6	0.39 ± 0.07	−9.1 ± 2.3	96.8 ± 3.4	96.8 ± 3.4
RG503	30	474.2 ± 57.2	0.46 ± 0.14	−9.4 ± 2.7	57.9 ± 1.9	96.5 ± 3.2

## Supplementary Materials

The following are available online at https://www.mdpi.com/2079-4983/10/2/26/s1, Figure S1: ^1^H NMR of 4-(2-hydroxyethoxy)benzaldehyde (**2**). Figure S2: ^13^C NMR of 4-(2-hydroxyethoxy)benzaldehyde (**2**). Figure S3: ^1^H NMR of (**3**) (3, 5-dimethyl-1H-pyrrol-2-yl)-(tetramethyl-4, 4-difluoro-4-bora-3a, 4a-diaza-indacene) methyl] phenoxy] ethanol. Figure S4: ^13^C NMR of (**3**) (3, 5-dimethyl-1H-pyrrol-2-yl)-(tetramethyl-4, 4-difluoro-4-bora-3a, 4a-diaza-indacene) methyl] phenoxy] ethanol. Figure S5: ^19^F NMR of (**3**) (3, 5-dimethyl-1H-pyrrol-2-yl)-(tetramethyl-4, 4-difluoro-4-bora-3a, 4a-diaza-indacene) methyl] phenoxy] ethanol. Figure S6: ^1^H NMR of (**4**) tetramethyl-4,4-difluoro-4-bora-3a,4a-diazaindacene)methyl]phenoxy]ethyl (4-nitrophenyl) carbonate. Figure S7: ^13^C NMR of (**4**) tetramethyl-4,4-difluoro-4-bora-3a,4a-diazaindacene)methyl]phenoxy]ethyl (4-nitrophenyl) carbonate. Figure S8: ^19^F NMR of (**4**) tetramethyl-4,4-difluoro-4-bora-3a,4a-diazaindacene)methyl]phenoxy]ethyl (4-nitrophenyl) carbonate. Figure S9: ^19^F NMR of (**4**) tetramethyl-4,4-difluoro-4-bora-3a,4a-diazaindacene)methyl]phenoxy]ethyl (4-nitrophenyl) carbonate. Figure S10: ^1^H NMR of the fluorescent tobramycin derivative (**B**). Figure S11: Mass spectrometry data for the fluorescent tobramycin derivative (**B**). Figure S12: Mass spectrometry data for the fluorescent tobramycin derivative (**B**). Figure S13: Elemental composition report for the fluorescent tobramycin derivative (**B**). Figure S14: Effect of the molar ratio of AOT:tobramycin on the extraction of tobramycin into dichloromethane. Figure S15: Tobramycin calibration curve in the absence/presence of AOT.
