# Supplementary material for: Formulation of Antimicrobial Tobramycin Loaded PLGA Nanoparticles via Complexation with AOT
Source: J Funct Biomater. 2019 Jun 13;10(2):26. doi: 10.3390/jfb10020026 (PMC6617385; doi:10.3390/jfb10020026)
Supplement: Supplementary file 1 [file jfb-10-00026-s001.pdf]

Journal of Functional Biomaterials

Supplementary Information

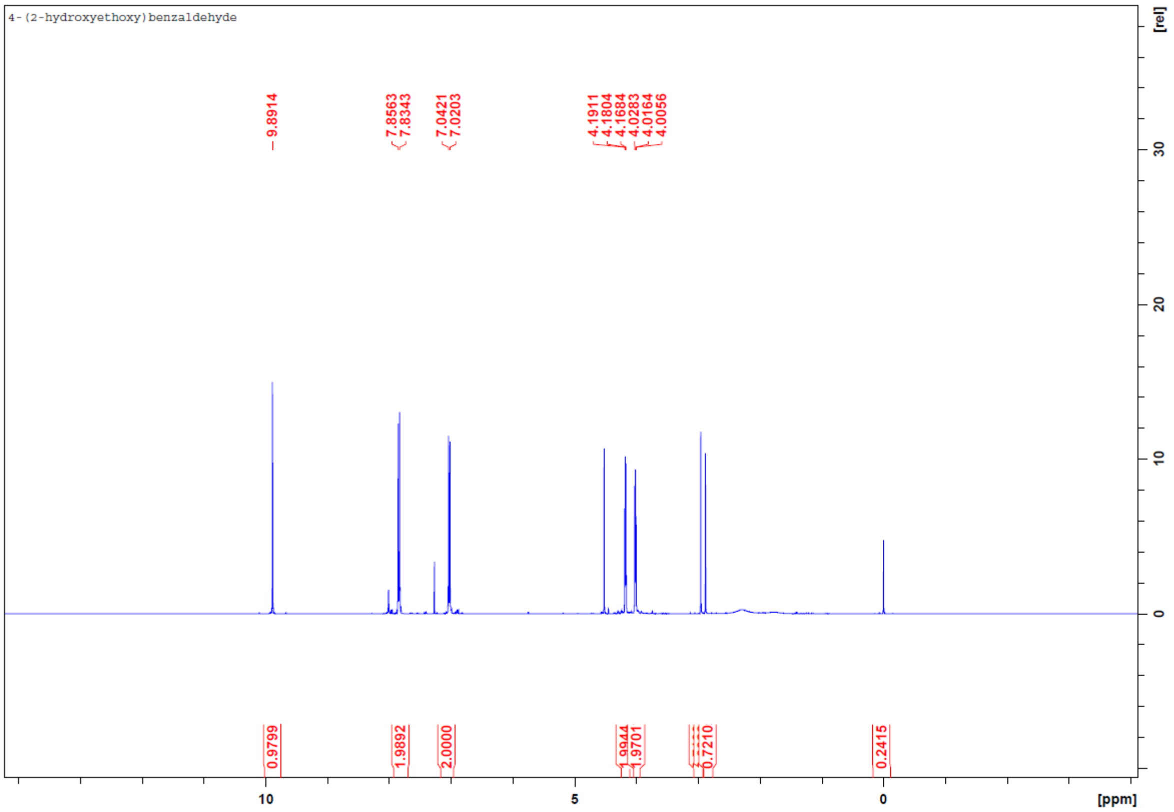

Figure S1. ^{1}H NMR of 4-(2-hydroxyethoxy)benzaldehyde (**2**).

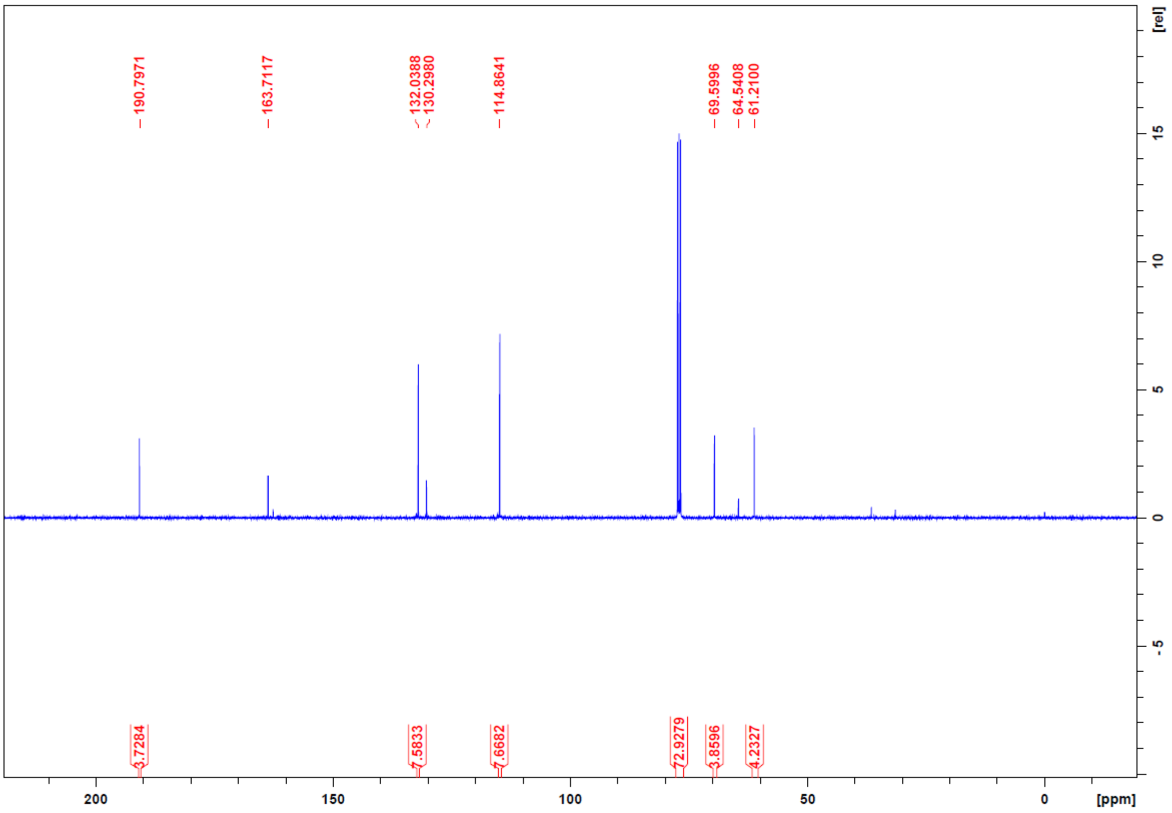

Figure S2. ^{13}C NMR of 4-(2-hydroxyethoxy)benzaldehyde (**2**).

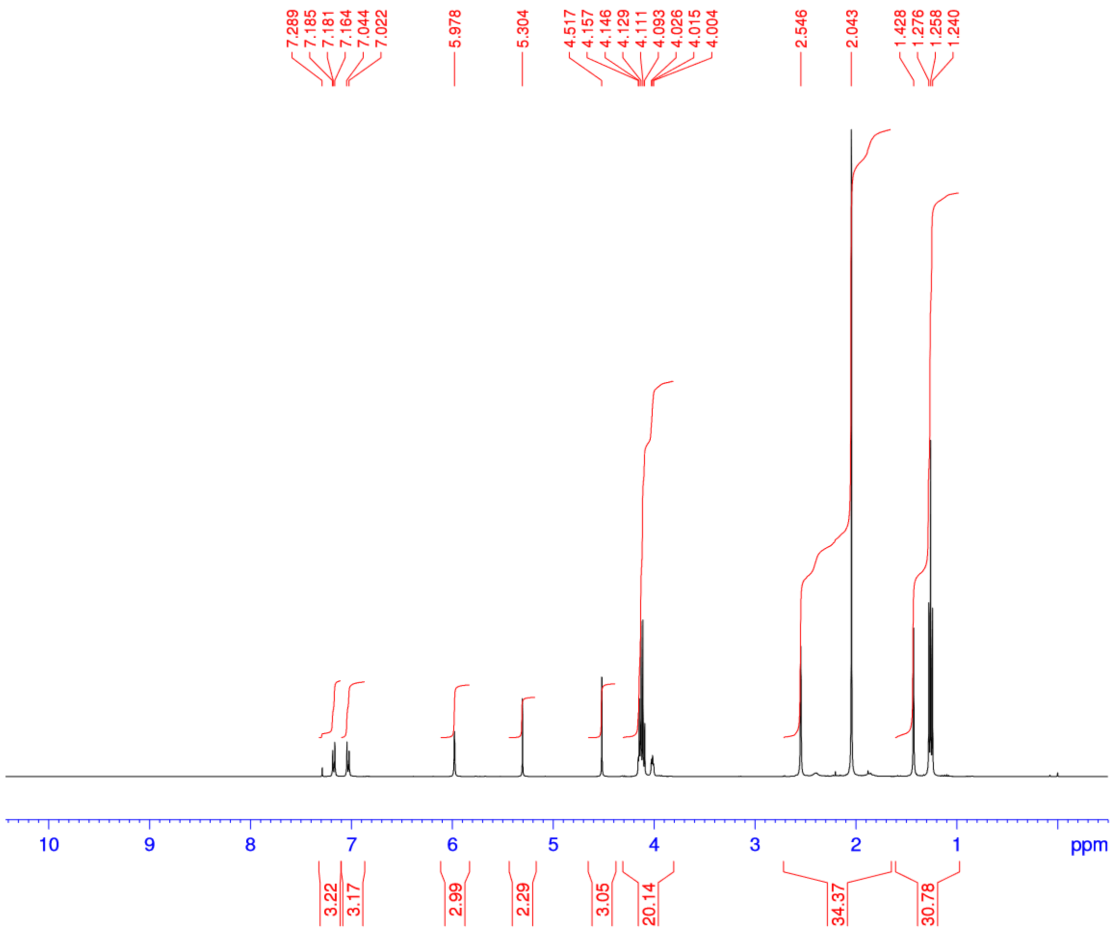

Figure S3. ^{1}H NMR of (**3**) (3, 5-dimethyl-1H-pyrrol-2-yl)-(tetramethyl-4, 4-difluoro-4-bora-3a, 4a-diaza-indacene) methyl] phenoxy] ethanol.

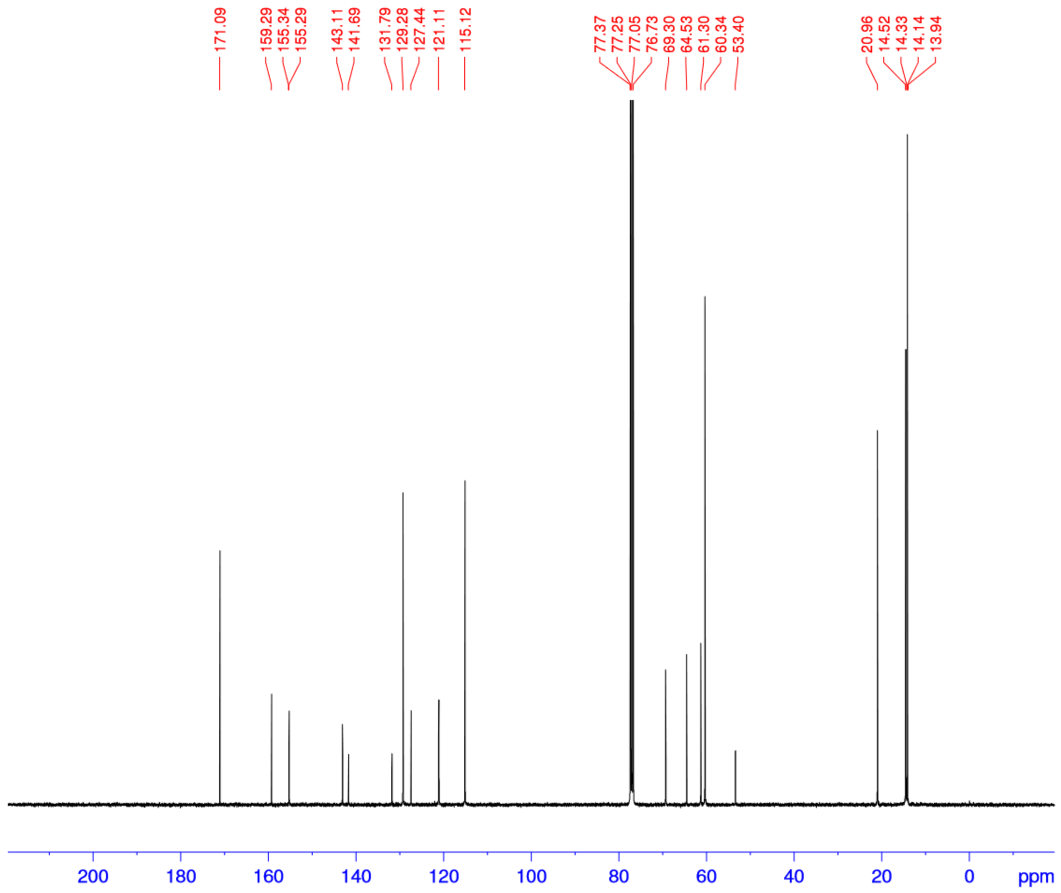

Figure S4. ^{13}C NMR of (**3**) (3, 5-dimethyl-1H-pyrrol-2-yl)-(tetramethyl-4, 4-difluoro-4-bora-3a, 4a-diaza-indacene) methyl] phenoxy] ethanol.

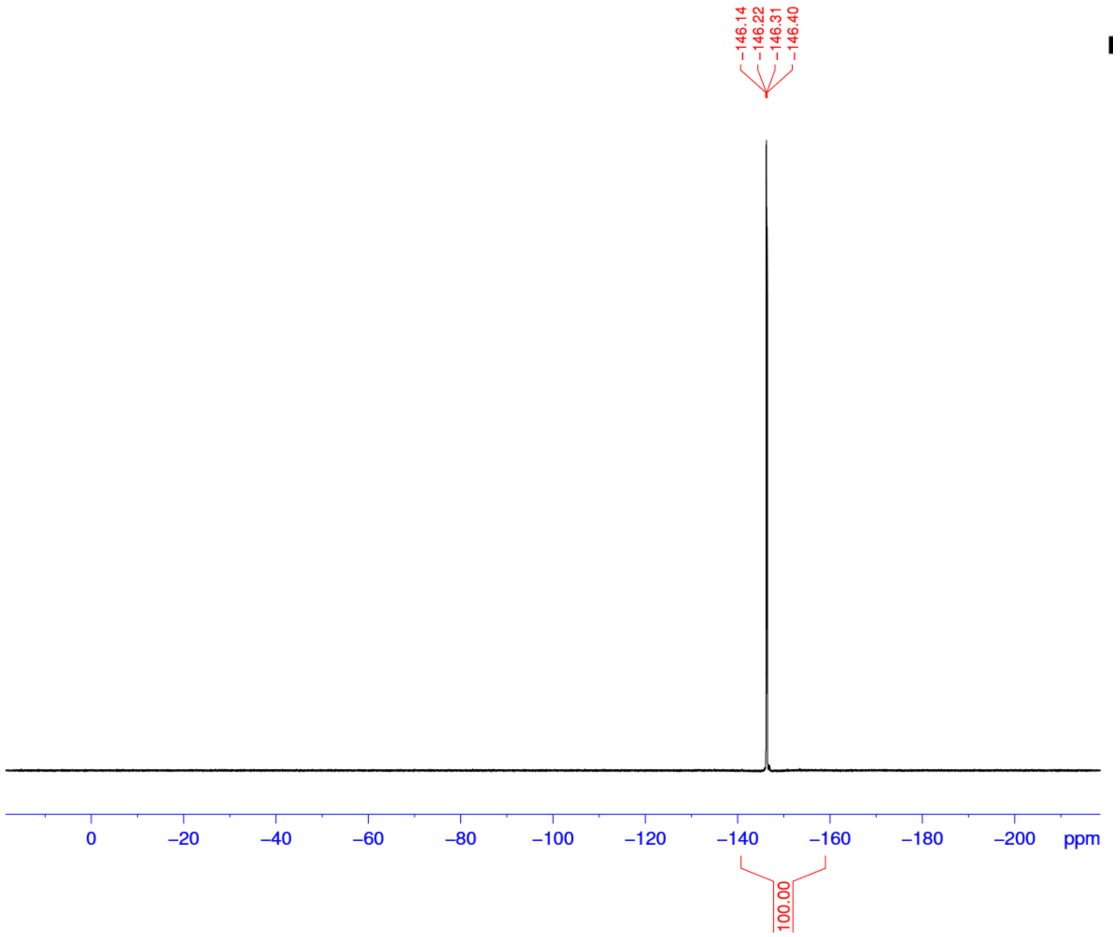

Figure S5. ^{19}F NMR of (**3**) (3, 5-dimethyl-1H-pyrrol-2-yl)-(tetramethyl-4, 4-difluoro-4-bora-3a, 4a-diaza-indacene) methyl] phenoxy] ethanol.

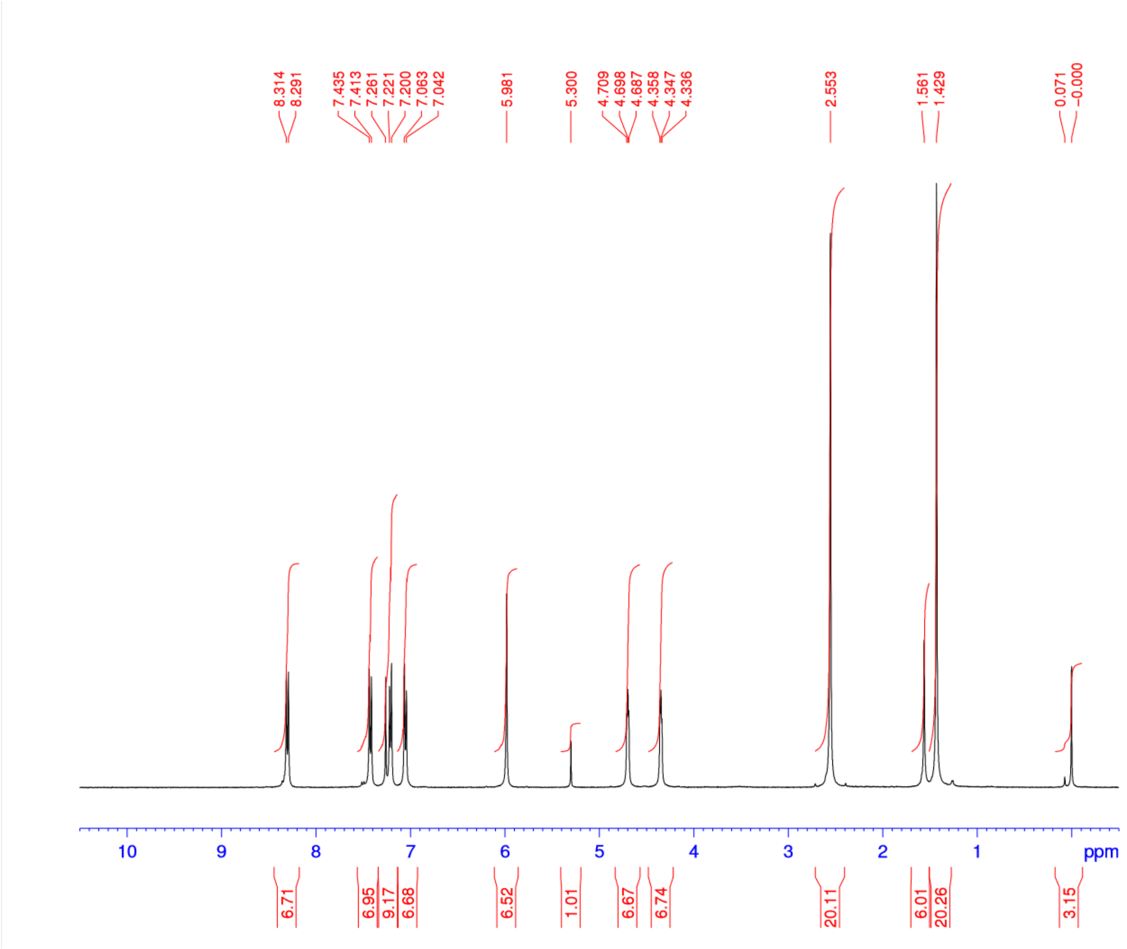

Figure S6. ^{1}H NMR of (**4**) tetramethyl-4,4-difluoro-4-bora-3a,4a-diazaindacene)methyl]phenoxy]ethyl (4-nitrophenyl) carbonate.

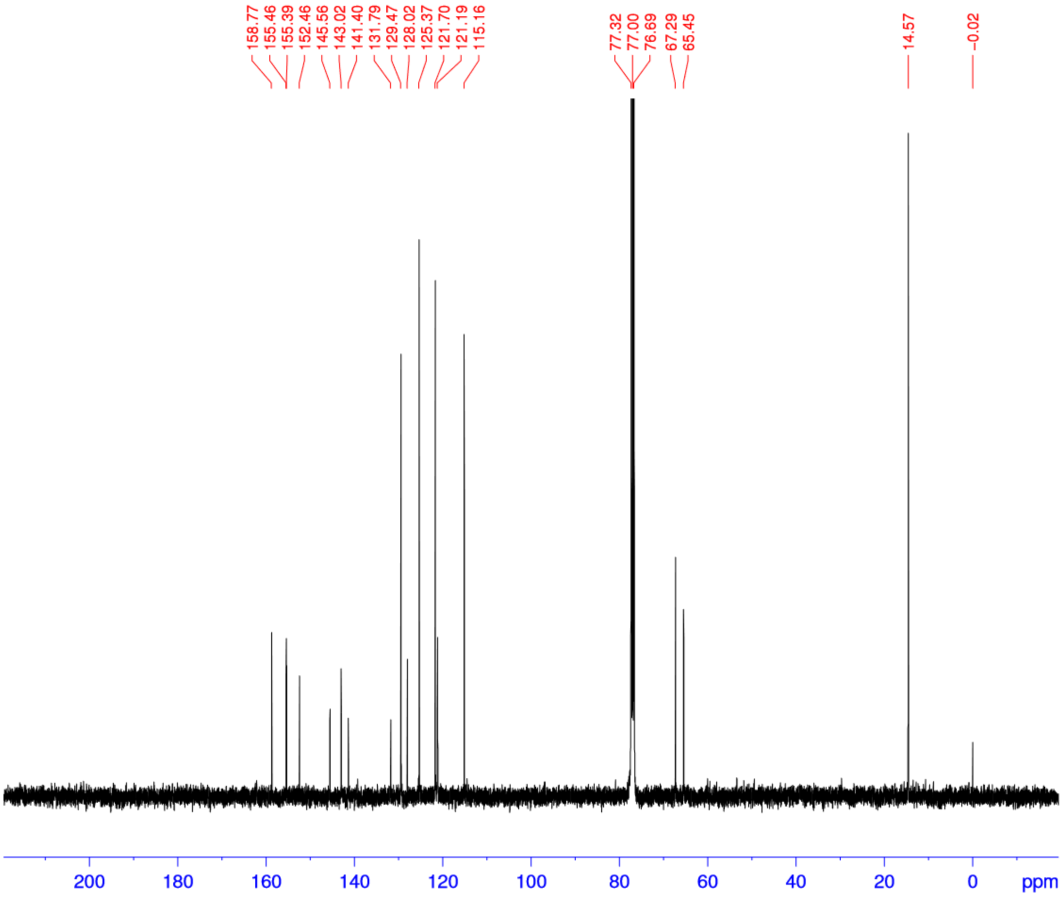

Figure S7. ^{13}C NMR of (**4**) tetramethyl-4,4-difluoro-4-bora-3a,4a-diazaindacene)methyl]phenoxy]ethyl (4-nitrophenyl) carbonate.

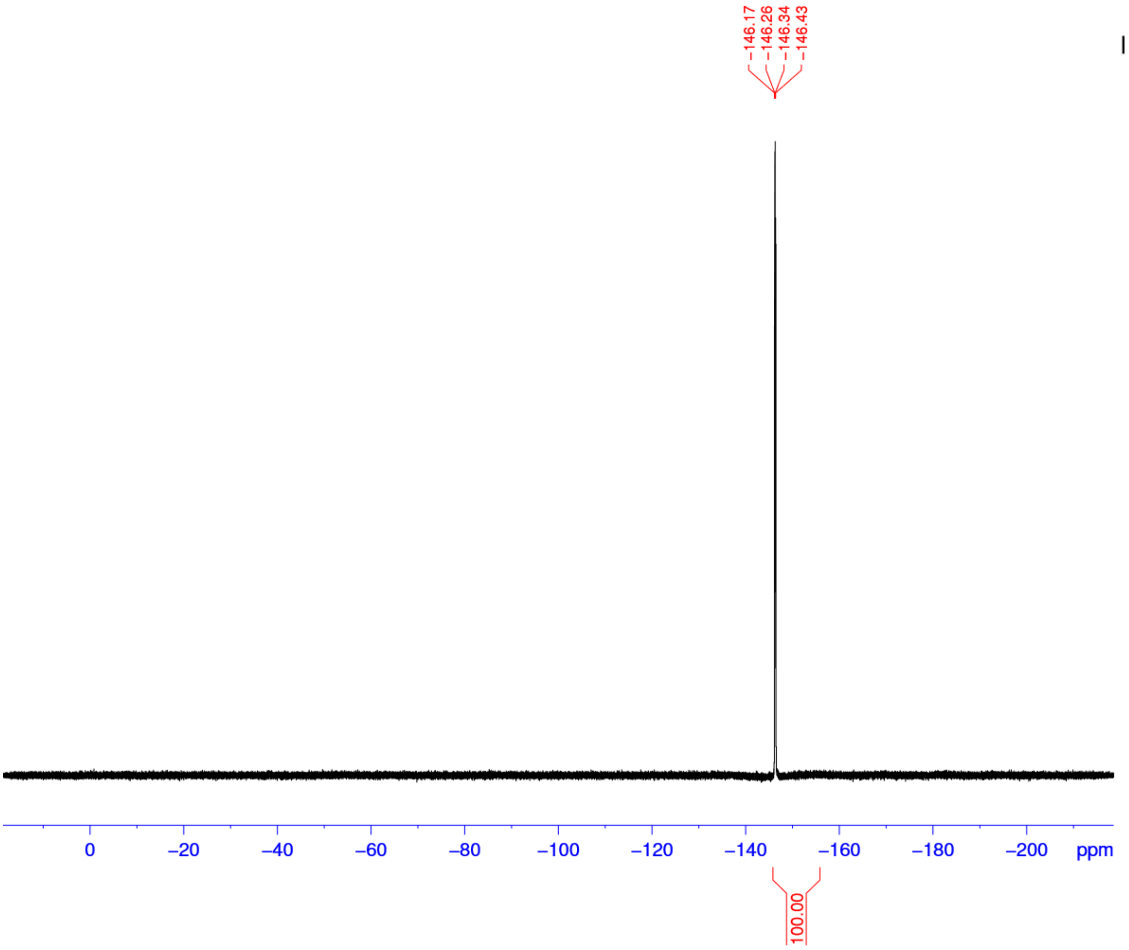

Figure S8. ^{19}F NMR of (**4**) tetramethyl-4,4-difluoro-4-bora-3a,4a-diazaindacene)methyl]phenoxy]ethyl (4-nitrophenyl) carbonate.

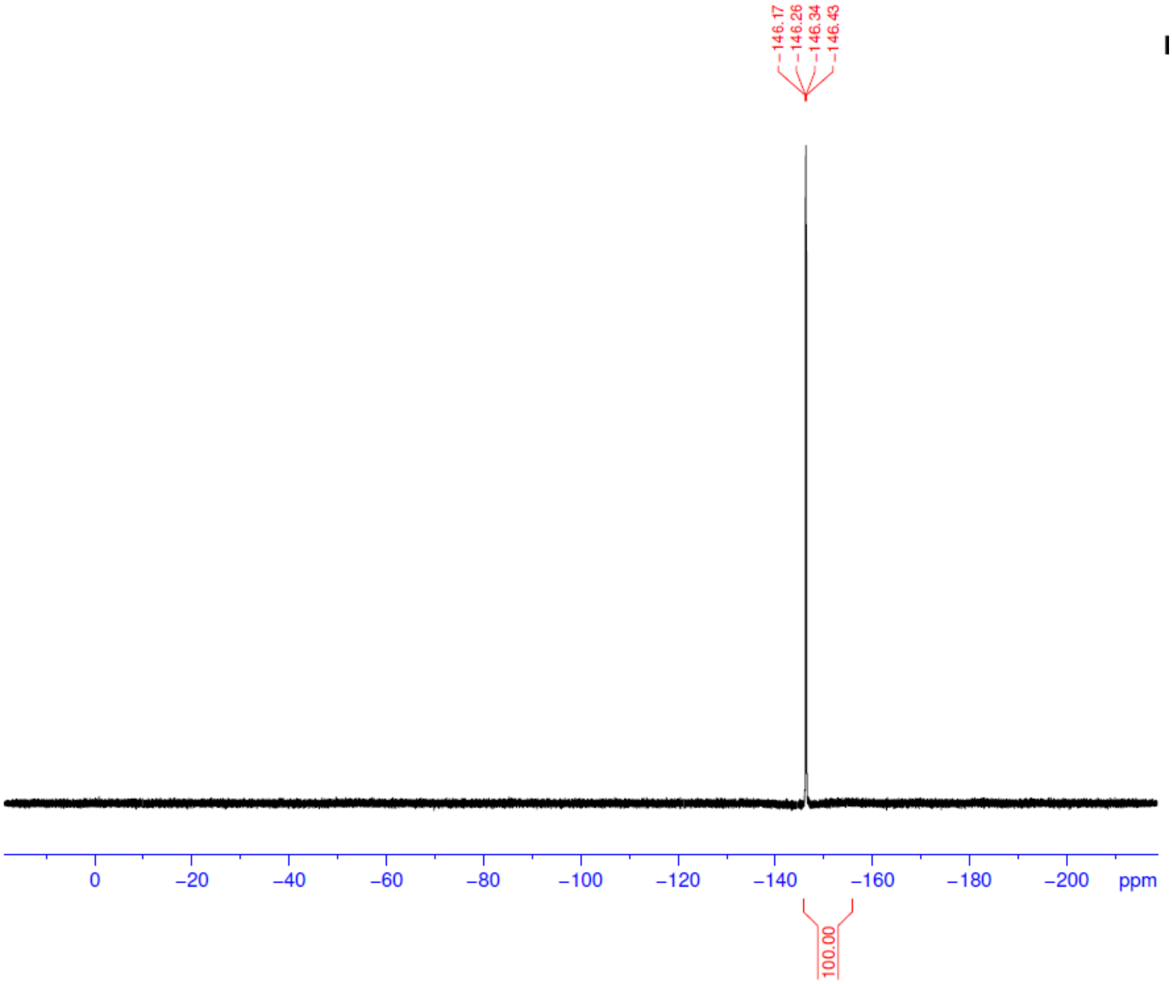

Figure S9. ^{19}F NMR of (**4**) tetramethyl-4,4-difluoro-4-bora-3a,4a-diazaindacene)methyl]phenoxy]ethyl (4-nitrophenyl) carbonate.

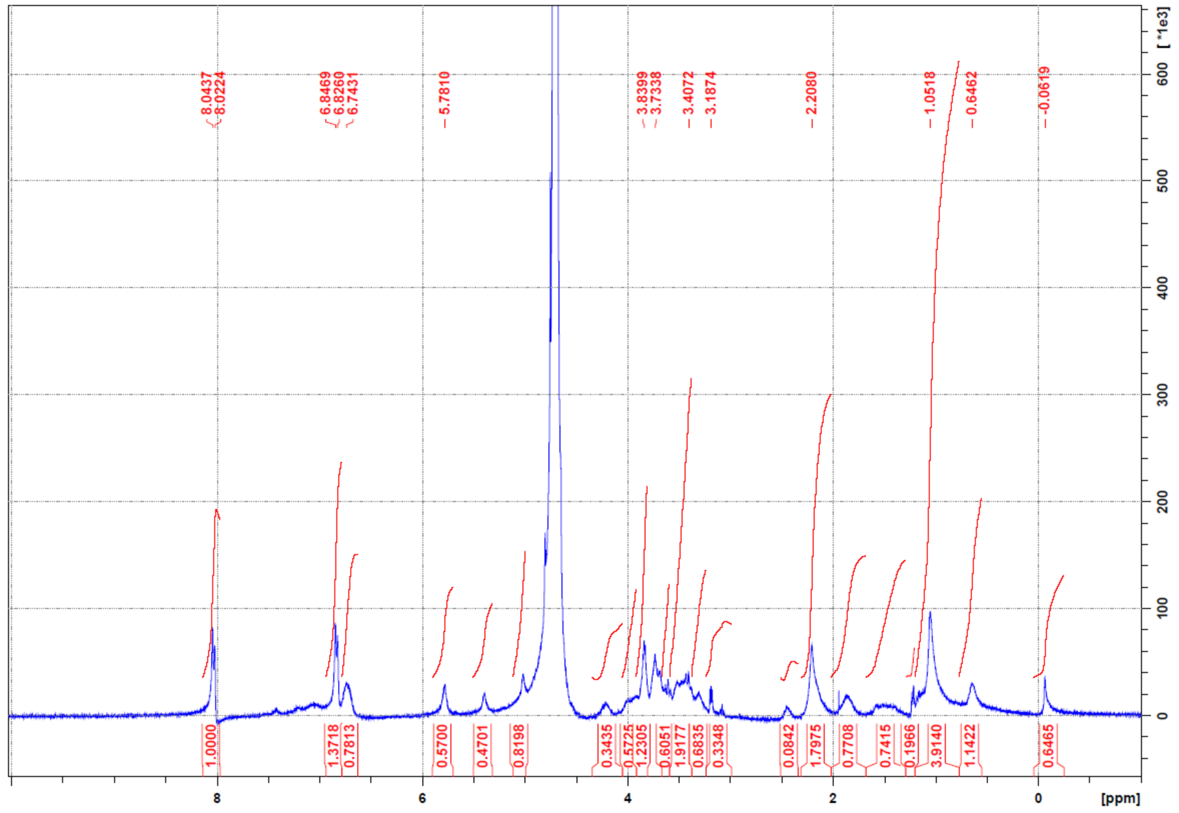

Figure S10. ^{1}H NMR of the fluorescent tobramycin derivative (**B**).

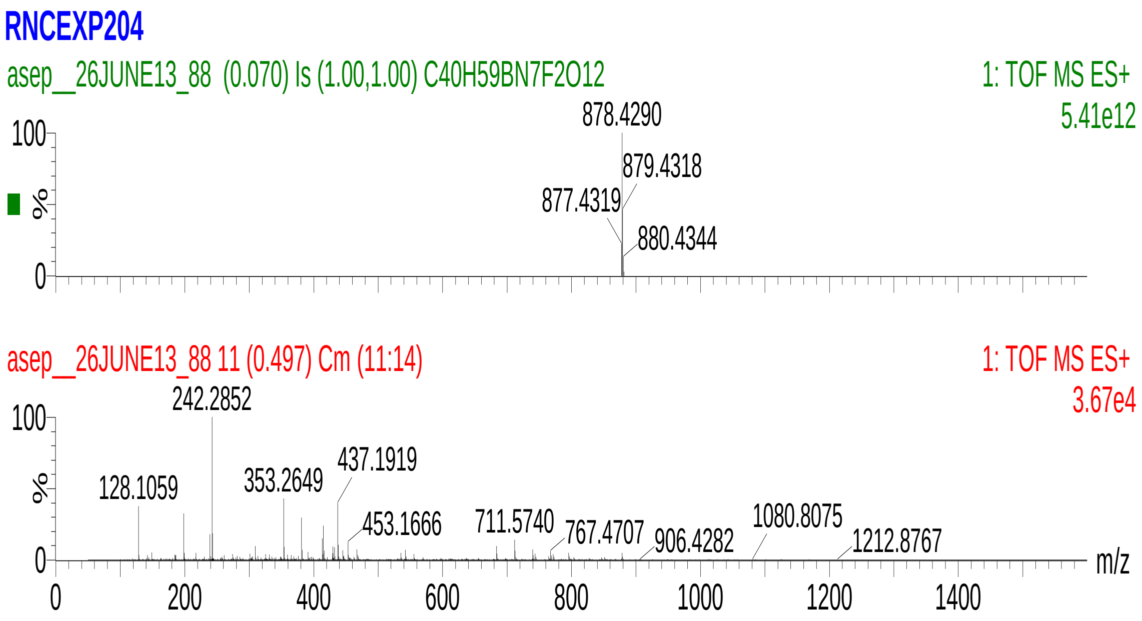

Figure S11. Mass spectrometry data for the fluorescent tobramycin derivative (**B**).

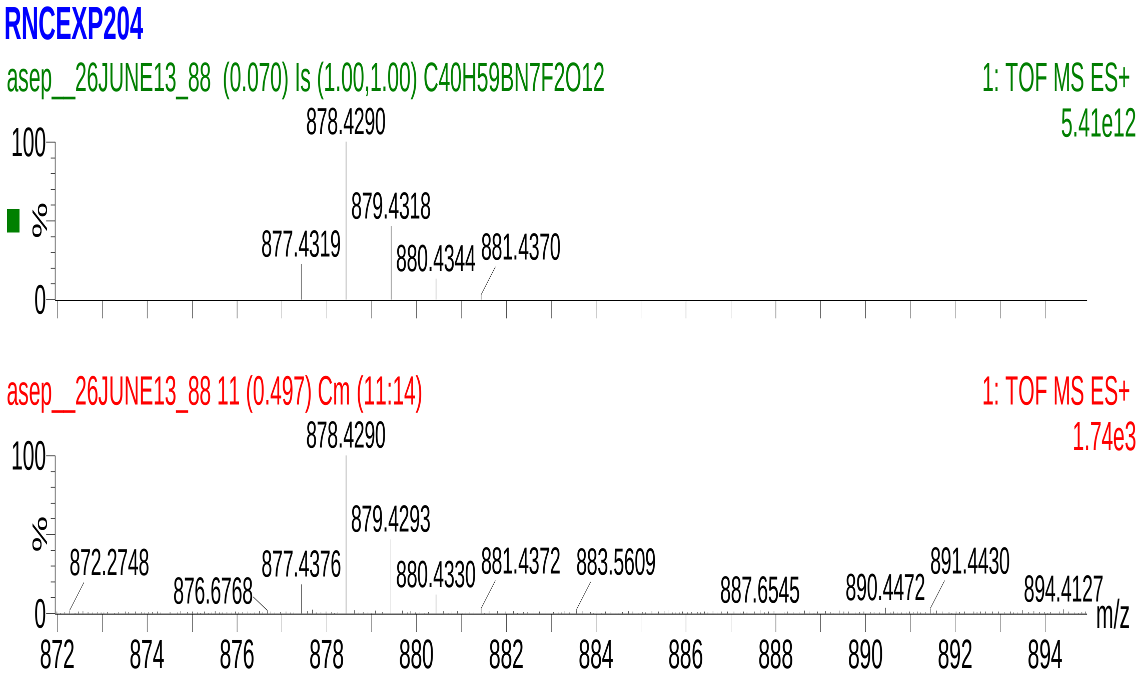

Figure S12. Mass spectrometry data for the fluorescent tobramycin derivative (**B**).

Elemental Composition Report

Single Mass Analysis

Tolerance = 2.0 PPM / DBE: min = -1.5, max = 50.0

Element prediction: Off

Number of isotope peaks used for i-FIT = 4

Monoisotopic Mass, Even Electron Ions

955 formula(e) evaluated with 2 results within limits (all results (up to 1000) for each mass)

Elements Used:

C: 0-80 H: 0-120 B: 0-1 N: 4-7 O: 0-12 F: 2-2

Minimum:				-1.5		
Maximum:		5.0	2.0	50.0		
Mass	Calc. Mass	mDa	PPM	DBE	i-FIT	Formula
878.4290	878.4283	0.7	0.8	14.5	1.6	C40 H59 B N7 O12 F2

Figure S13. Elemental composition report for the fluorescent tobramycin derivative (**B**).

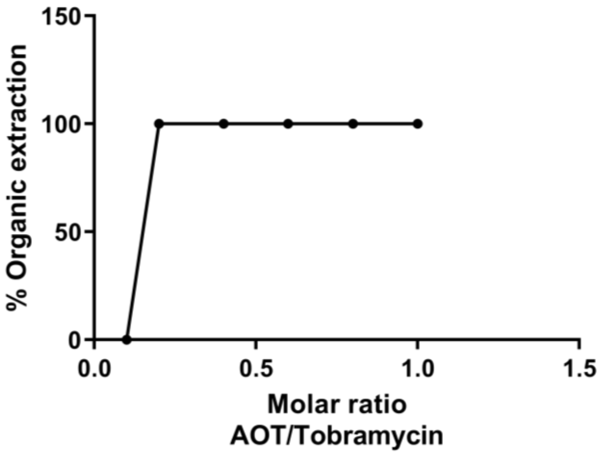

Figure S14. Effect of the molar ratio of AOT:tobramycin on the extraction of tobramycin into dichloromethane.

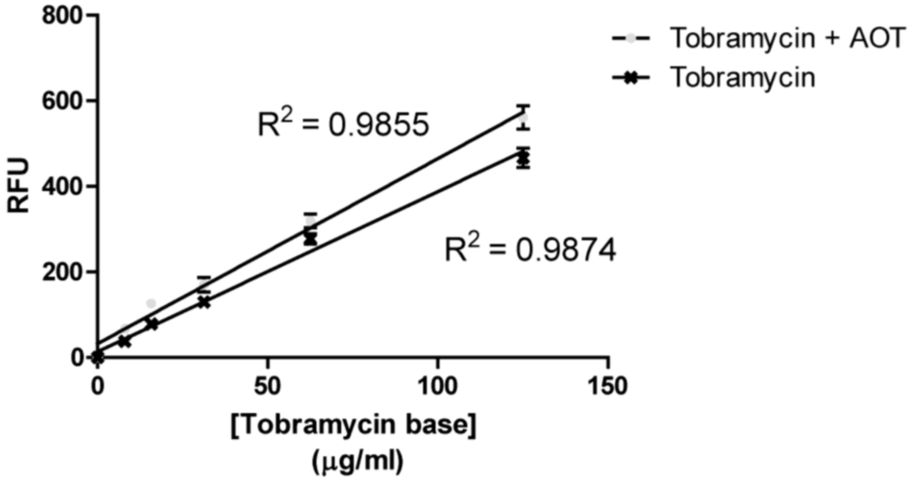

Figure S15. Tobramycin calibration curve in the absence/presence of AOT.
